# Relationship at work as a cause of occupational stress: the case of academic women in Vietnam

**DOI:** 10.1186/s13033-016-0078-2

**Published:** 2016-05-25

**Authors:** Le Van Thanh

**Affiliations:** University of KhanhHoa, 01 Nguyen Chanh, Nhatrang, Khanh Hoa Vietnam

**Keywords:** Grounded theory research, Vietnam, Occupational stress, Confrontation, Confucian culture

## Abstract

**Background:**

The present research paper aims to bring deeper understanding and insight to perceptions and experiences of occupational stress from relationships at work in the cultural context of Vietnam. The paper also examines differences in perceptions, experiences of occupational stress from this problem and makes a comparison with perspectives in other cultures.

**Methods:**

Grounded theory approach is used to study occupational stress by collecting data from in-depth interviews with 42 academic women employed at Vietnamese higher education institutions to understand the meaning, the nature and source of the occupational stress from relationships at work they experience and the impact of occupational stress on their lives.

**Results:**

Cultural factors play an important role in occupational stress. Cultural factors such as power distance and hierarchy influence perception, experiences of occupational stress and the ways occupational stress is responded to. The Vietnamese context differs from other cultural contexts in the range of factors perceived as stressors for Vietnamese women.

**Conclusion:**

This paper is the first grounded theory study of occupational stress among women academics in Vietnam that determines that the natural of the relationship at work play an important role in how women understand and respond to occupational stress and supports the growing evidence that occupational stress is common, global but different in other cultures.

## Background

This research aims to explore experience of occupational stress, originating from relationships at work, among Vietnamese academic women in higher education in a Vietnamese cultural setting that is chose as an example of Vietnamese cultural influence. The focus on only women academics has been decided upon because Vietnamese women in the academic work setting are good representation of the values that are under the influence of Confucius ethics. They provide the opportunities to uncover the influence of Vietnamese culture on occupational stress experienced by women in the academic field, the opportunities to compare to the Vietnamese women’s experience and perception of occupational stress with that of their counterparts in other cultures, the opportunity to discover grounded data needed to develop an understanding of occupational stress in the academic environment and useful information that would help design the support system needed to encourage more woman into the noble profession of education.

Of particular concern to the present study is the relative lack of research focused on women academics in Vietnamese culture. Since occupational stress is not unique to women, the research draws special attention to the very particular problems of women academics in Vietnamese cultural context that can provide a solid basis for further study of women’s occupational stress experiences from their individual perspectives.

The stress experienced by staff in higher education as an occupation is widely recognized by several studies initiated in Western countries to address its causes [43] and the academic profession is under more occupational stress than ever before [1, 20]. Women are now increasingly represented among staff in higher education [40]; for example, The representation of women in higher education is relatively low at 44.4 % of academics [30] and Western studies indicate that gender plays a part in academics’ experience of occupational stress [6]; and significant stressors in academic life that have been identified are: poor relationships (lack of support, conflicts), poor management,

Occupational stress appears to be a feature of occupational life for academics [17] and higher education institutions seem to be a male-dominated working environment. Male-dominated work cultures can place women under high levels of occupational stress compared with men [10, 41, 43]. Gender is a socially constructed category and there are different expectations for men and women in society [2] which have an influence on their experiences of occupational stress [23]. Vagg et al. [42] argue that gender is a key determinant of occupational stress reflecting a wide acceptance of this viewpoint; the effects of gender on occupational stress have been examined in many studies [7, 14, 23, 24, 29, 33, 34].

A grounded theory approach is chosen and used relying on in-depth interviews to collect and analyze data; as the concepts of occupational stress are transferable from culture to culture [35] and in general, most research on occupational stress have used Western-based approach, but these have proved to be unconvincing in regard to documenting the phenomenon accurately and the tools can not explain adequately the features of occupational stress [18, 27]. By exploring the phenomenon of occupational stress among academic women in Vietnamese culture, it is anticipated that the research findings would be explained to improve understanding of this phenomenon and provide new knowledge about occupational stress in Vietnamese cultural specific context and to provide a foundation for future cross-cultural research on the phenomenon. The researcher’s experiences of working with academics in Vietnamese higher education has been identified as the ambition for pursuing study in this field and these experiences also contribute to the research.

## Methods

This research adopted a grounded theory approach which framed occupational stress as being a phenomenon constructed that was useful in addressing the gap in occupational stress studies by focusing on women academics in Vietnamese culture. Face-to face interviews were an appropriate methodology to capture and explore experiences of occupational stress because it captured a particular context and made space for representations of various voices with a stake in the study [12] and it tapped into the unique kind of knowledge that was communicated through interviews [25]. Ethical conditions were considered whilst conducting in-depth interviews. The study applied for ethics approval (Form C) from the Research and Ethics Committee of Curtin University prior to the beginning of the study and continued for the duration of the study.

The thesis investigator used interviews about women academic participants’ experiences of occupational stress as the primary source for exploring and understanding the studied phenomenon from the perspective of the participants experiencing it. Nvivo computer software program was also selected as an assistant tool from the start of the thesis, through to data collection and analysis process and the writing of the final document [37]. This program enhanced the validity of the research and allowed the investigator to build the study findings and thoughtful interpretation closely with data analysis [5]. The research met the criterion of relevance of grounded theory research on a social phenomenon because it addressed women’s concerns with the phenomenon and strived to answer the questions that could improve conditions of their working and lives.

### Sampling

The research data were collected over 5 months from 8/2010 to 12/2010 at six higher education institutions in NhaTrang city, Vietnam which were invited to participate in the study with the permission from the Principals. These institutions comprise of 3 universities and 3 colleges, in which there are 5 public and 1 private institutions; 3 multiple-disciplined and 3 mono-disciplined institutions. These selected institutions have substantially different management (public vs. private), human resource characteristics and practices (multiple- and mono-disciplined). Selecting a variety of types of institutions was considered to avoid homogeneity among them by choosing various fields of higher education: Education, Health, Technology, Arts and Culture. The institutions undertaken in this research were considered to be the optimal number in order to ensure quality of research data, a sufficient collection of data and participant selection in the form of theoretical sampling.

The participants in this research were full time, female employees who belonged to the career staff of one of the six institutions and who were responsible for teaching and research in their institutions. This sample was selected by the using theoretical sampling technique advocated by Creswell [9]. Theoretical sampling is the “sampling on the basis of concepts that have proven theoretical relevance to the evolving findings” [38]. The number of participants recruited at each stage is determined by the data analysis process following the previous stage until theoretical saturation of data is achieved [11]. Corbin and Strauss [8] assert that saturation can be achieved with between 10 and 60 participants. This view is taken in this research because fewer than ten renders it almost impossible to interpret the studied phenomenon completely; more than sixty makes it difficult to cope with the time taken to gather the information. The selection was designed to collect data from a heterogeneous sample of subjects.

### Data analysis

The research method chosen for the current study that focuses on the investigation of occupational stress among academic women in Vietnam is grounded theory approach. The aim of this process is to look for core categories and its relations with other categories between various themes that emerge as the result of the data collection and analysis in order to answer the research questions. Data collection and analysis occurred simultaneously after the first interview. Constant comparison and coding of the data began from the second interview, with categories being progressively refined as more data is collected. In the section, theoretical sensitivity and the process of three stages of coding and constant comparison are explained and described.

#### Coding

##### Open coding

In the open coding, initial analysis of raw data was taken at descriptive level in which data comprising participants’ perspectives of occupational stress from relations at works were constantly compared and broken down into as many codes as possible stored in nodes. Many codes were subsumed at a conceptual level to establish labeled concepts in nodes which were either free standing (free nodes) or grouped together into a node structure (tree nodes); and many code words could become concepts, and categories. The constant comparative method was applied to reduce the number of codes as concurrent analysis of subsequence data modified and supported and to group concepts with common meaning together to construct and develop lists of categories.

##### Axial coding

Following the open coding stage, axial coding was used to cluster the data by making relations of categories to its subcategories and between the categories. The identified categories were compared to one another and to the phenomenon to ensure they were exclusive. During this stage, new data from subsequent transcripts were also compared constantly in order to identify the connections between the categories and their subcategories and among categories; for example, categories: “*relationships with seniors”* and “*relationships with managers*” were found to have relations with category “*relationships at work*” which was a cause of occupational stress, described in Table 1.

**Table 1:** Relations between categories

Code (participant’s words)	Relations between categories
Relationships at work with my seniors and managers are a great source of occupational stress (GCD)	

##### Theoretical coding

Following gathering and analyzing the data, the categories were integrated to shape findings of the investigated phenomenon by theoretical coding, which was the process of conceptualizing, integrating the findings by identifying the core category: *occupational stress* and relating subcategories around the core category by using paradigm: conditions, context, strategies and consequences [39]. For example, subcategories “*relationships with colleagues*” and “*relationships with managers*” were linked to the category “*relations at work*” that was a cause (condition) of occupational stress from which women academics suffered. Different categories were compared and tested by extant and new data.

Constant comparisons of incidents from subsequent interviews in the stage to established concepts and categories derived from the axial coding were continued to fill categories that required further refinement and/or development, to validate the relationships against the data and to cluster to create integrated and saturated categories [9].

In terms of the paradigm model espoused by Strauss and Corbin [39] and Creswell [9], the relationships between categories and subcategories were implicated by determining a category in terms of *a central phenomenon* (i.e., the core category about the phenomenon—occupational stress), *causal conditions* (i.e., categories of conditions that led to the development of the phenomenon, such as relationships with the older was one of the causes that led to occurrence of occupational stress), *the context* (i.e., under conditions (cultural) from which occupational stress had been developed and influenced) which it was embedded, *strategies* (i.e., the action strategies developed to respond to the phenomenon, such as avoidance) and *consequences* (i.e., the results of the strategies) for the phenomenon.

## Results

Like other Confucian cultures, relationships played a crucial role in the Vietnamese setting. It was important for the Vietnamese to maintain and cultivate their relationships with people at work in order to have good opportunities and standing in the workplace, but the participants also reported that they really experienced stressful events in relationships at work:

Relationships make me uncomfortable and unhappy, I find frustration in these relationships and this is stress (VACD).

The superiors expected their subordinates to show being afraid and interested in them, through the presentation of gifts given manifests care. Occupational stress resulted from a number of features of the relationships at work. In some cases, the cause of occupational stress is poor knowledge of the other people in the unit or faculty that made poor relationships which hindered their career:

If I have good relationships, they help me handle my work quickly; if the relationships are not good, people make me difficult (QYT).

Relationships were an important source of concern for the participants, because they felt that they had to constantly achieve good relations and harmony in their working settings, even when they felt unhappy. Creating relationships with colleagues and achieving harmony was so difficult for some participants that it was stressful for them:

I do not know how to please everyone; maintaining harmony is too difficult. Stress from relationships with them is significant (PCD).

It is apparent that stress from relationships can occur because of the relationships with the older, superiors and peers at work as the following.

### Those with older colleagues

Many participants, including lecturers with management responsibilities, felt relationships with their colleagues the most stressful, especially relationships with older counterparts due to the emphasis on hierarchy in the Vietnamese culture:

Hierarchy in relations with colleagues creates difficulties … which are how to use words in relationships with older colleagues (KVH).

The Vietnamese people acknowledge that opinions of the older are always true, more powerful and better than that of the young “an egg (young) is never smarter than a duck (old)”, it means that age carries knowledge, wisdom and experience and respect for the senior is a cardinal factor in Vietnamese society; as a participant complained:

In Vietnamese culture, older people often assume that their opinions always are right (TVH).

Not only academics but also both lecturers and managers felt building and maintaining harmonious relationships with older staff members to be very stressful, regardless of their education backgrounds and management positions, as a woman who is both a lecturer and a leader of the teaching area explained:

I am modest in relations with the older and not aggressive as they impose on me… because of hierarchical relationships; moreover I’m younger; although they have lower degrees (HTBD).

To keep good relationship with the older staff members, according to them, is beneficial for academics’ long work life. Therefore, participants are to respect and fear having conflicts with the senior ones:

I am mostly afraid of relationships with colleagues, so I always try to maintain these relationships, and I do not make them offended. If someone is only interested in short-term benefits, they will lose the relationships (TVH).

For new and young women academics, they feel occupational stress as a result of having to comply with often ‘unreasonable and irrelevant’ requests often made by the senior and experienced lecturers:

In the first year at the college, I just did clerical work, so I was stressed; then I am a lecturer, but I am forced to run errands, hence I feel heavily stressful. I also respond to the older, but not strongly, I go home to cry and talk to my parents, I am unhappy with it (TAYT).

For female academics with management responsibilities, they felt that they had power stipulated by the university’s rules and regulations. However, the older colleagues had power entrusted by the Vietnamese society, as a participant explained:

In relationships, generally speaking there are ranks and management positions. Degrees and expertise have created hierarchy and power; or management and ages also are hierarchical and powerful (LYT).

Young academic women commented on the lack of cooperation and on dissatisfaction with the level of collegial input and contact. They faced these problems and challenges at their work places and this made so of them so stressful that they said they wanted to leave their jobs. The following quote illustrates some of their frustration:

To overcome occupational stress from relationships with the older makes me easy to give up my job, I want to change the job that suits me better (XTBD).

They showed the comfort and warmth at work, but they were not satisfied, and these relationships inhibited development of the young:

I used to be inhibited when experienced people don’t have the same expertise, but they did not give me any chance to express (HCD).

These quotes demonstrate that the younger are stressed from the relationships with the older colleagues because of the Vietnamese cultural emphasis on of hierarchy, whereas participants who are the oldest and older than many members in their units and faculties do not find relationships with younger staff members stressful:

I’m not stressed from ages, because I’m older than many members of the unit. But older people have more limitations as they are not smart in their operations; my advantage is to have more practical experience than that of younger people. I’m older, so I’m not more active (HCD).

**Fig. 1:**
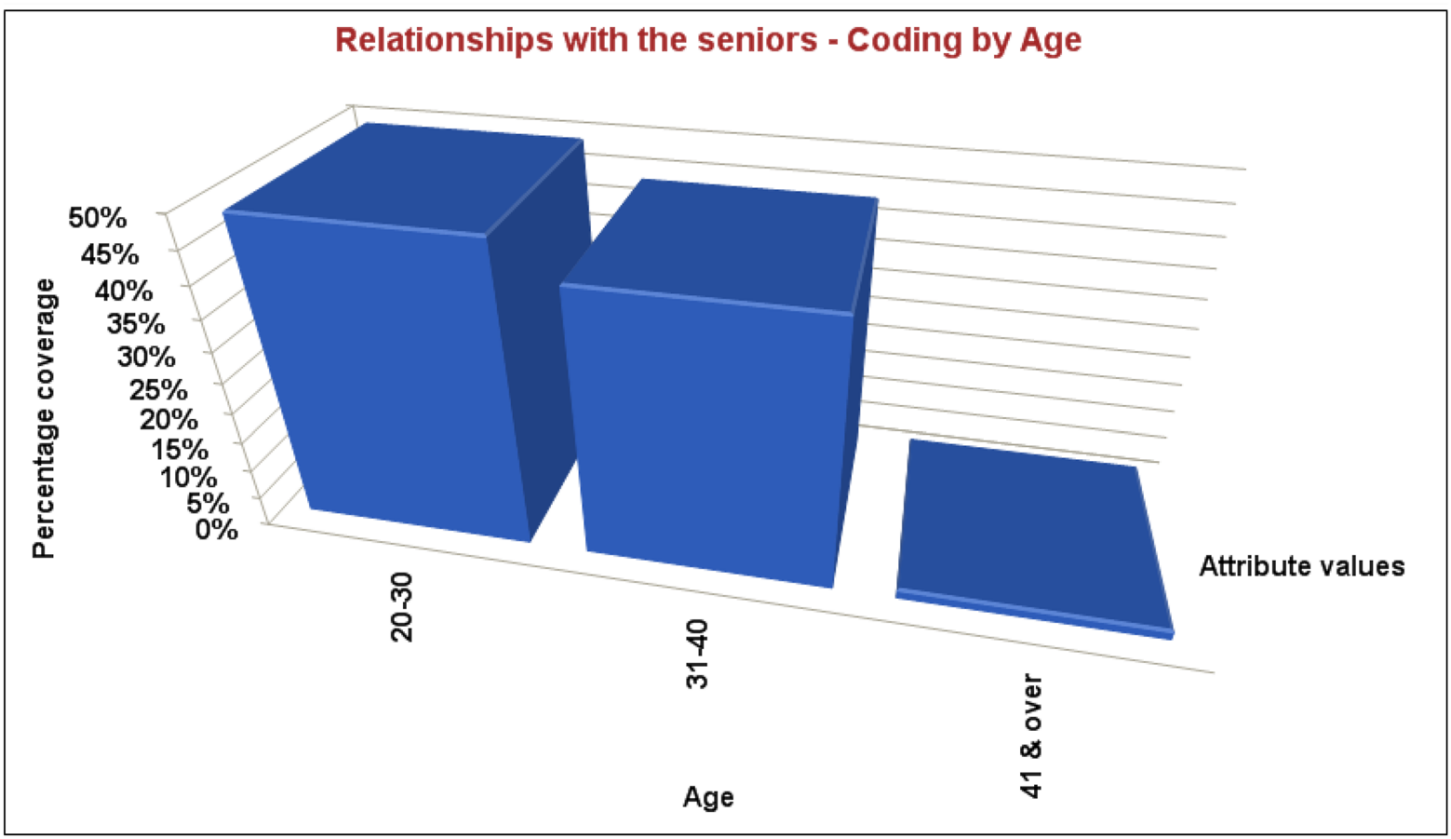
OS had a reverse relationship with age

As can be seen in the Fig. 1, OS had a reverse relationship with age. It was found that younger academic women (between 20 and 40 years old) suffered OS more than the older women (over 41):

### Those with superiors

The experiences of the participants shed light on how Vietnamese academic women suffer OS as a result of their relationships with older and more senior colleagues. Relationships at work between superiors and subordinates are affected by power distance and hierarchy. They are identified as a salient and pervasive source of occupational stress by the majority of interviewees,

The emphases on power distance and hierarchy appear to have resulted in a climate in which occupational stress from these relationships was seen as inevitable in Vietnamese academic women’s work life, for example: “*Power distance from which I am stressed is great”* (DTDT).

Power distance and hierarchical relationships also create stress. Power between superiors and subordinates is very high and patriarchal, unlike relations among the western (DCD).

A social hierarchy and power distance were referred in the relationships in which superiors hold a right to instruct subordinates who are obliged to obey

For managing officers, we have to always obey to them, and rarely dare to speak against them (though I disagree with their assignment); I have to “flatter” them so that we get more support, tolerance and receive more work from them (KNT).

These factors were concerned with keeping a distance and respecting superiors, so that “*leaders have high power, so subordinates are very afraid of them…. Subordinates must respect their leaders*” (HNT). In perceptions of Asian people in general and of the Vietnamese in particular, these factors were concerned with attitudes toward not only ages but also ranks at work and social positions in the society, just like: “*there is power distance between managers and subordinates”* (VTDT); “*power distance between managers and lecturers is high, especially between lecturers and their female or/and old managers*” (TAYT), because in Vietnamese culture: “*managers are always right in all cases*” (DTDT), moreover “*role of leadership of Asian countries is very high, and leaders are giant persons*” (TCD).

Hierarchy in Vietnamese culture creates high power distance between superiors and staff: “*Power of managers is too great*” (TCD) leads to “*The managers refuse to listen to their subordinates*” (TCD) because “*Managers often keep their opinions in a very conservative view*” (CCD). In Vietnamese culture, it is also perceived that people in high positions have higher degrees and hence high level of knowledge:

They suppose that people with advanced degrees and profound knowledge must hold top management positions (TCD).

However in reality, those in powerful positions do not necessarily have the required educational attainment and competency, as she complained:

I’m under the authority of a person who I don’t admire and who has a low degree, bad way of communication, no talent, and no morality, so I feel that I am offended and humiliated by her.

Hierarchy in relationships in Vietnamese culture made them keep face of their old colleagues and harmony in these relationships, and even to maintain the harmony also created stress on them: “*hierarchical culture … creates stress because I am afraid of losing my relationships*” (VTDT), so that

I create harmony and friendly relations with my colleagues, especially the seniors who are relatively successful, but I am stressed from relationships with successful colleagues like me…Making harmony is too difficult and stress from relationships with them is substantial (PCD).

**Fig. 2:**
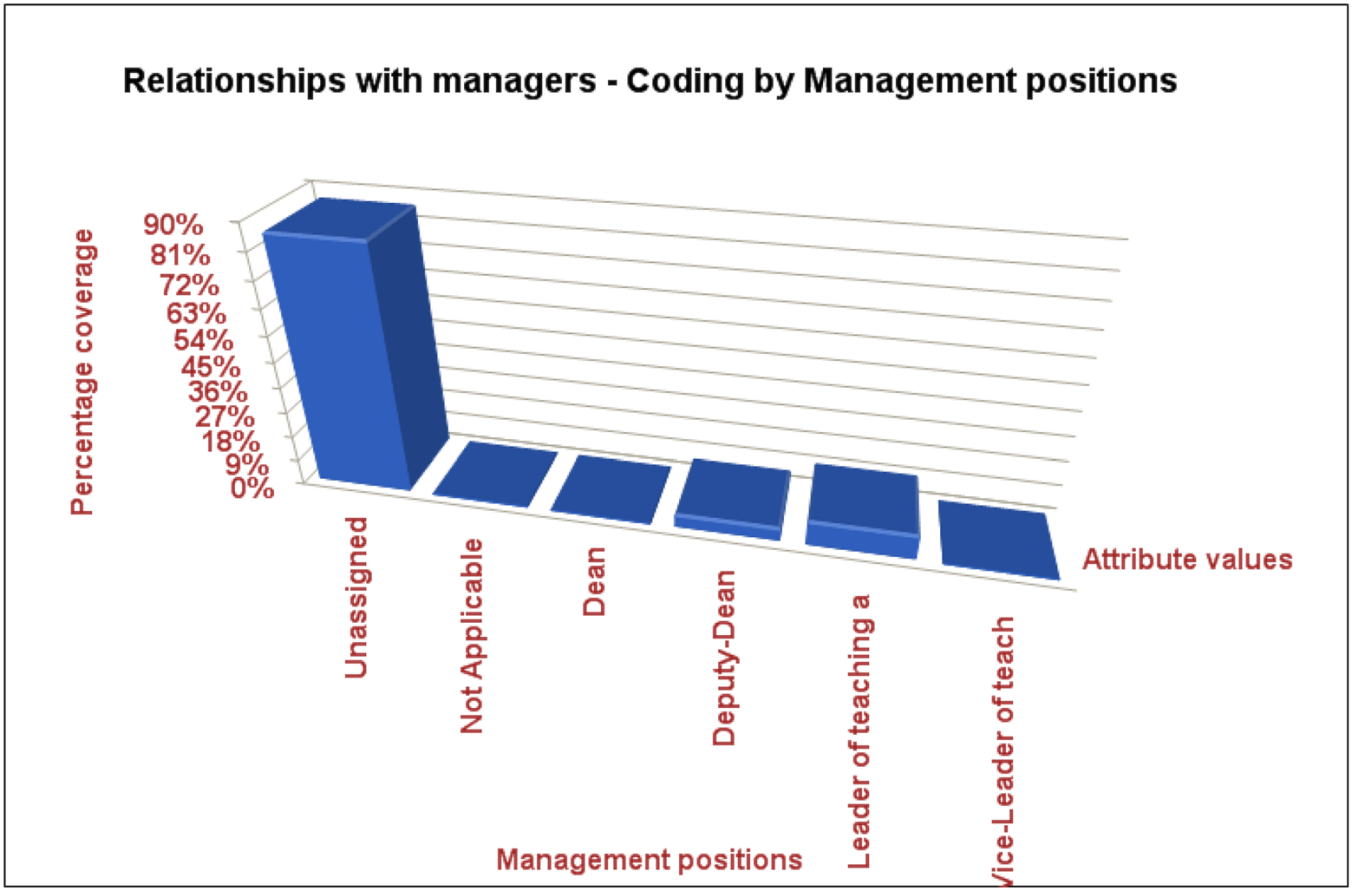
Indicates many academic women without managerial positions are stressed group in terms of their relationships with the superiors, compared with other groups of women academics who hold some managerial/leadership roles

The Fig. 2 indicates many academic women without managerial positions are stressed group in terms of their relationships with the superiors, compared with other groups of women academics who hold some managerial/leadership roles.

### Those with peers

Building and maintaining social networks and connections plays a vital role in the Vietnamese culture. The participants reported that this aspect of their culture affected their relationship with peers and found it stressful:

I am stressed from relationships with peers…. Stress from relationships with my colleagues is key…. I fear losing these relationships (QYT)

Factors influencing relationships with the peers are discussed in the following sections.

### Competition

Relationships with peers (i.e., those in the same age groups) are affected by competition within the group: *“I think competition is a source of stress, especially for staff with the same age and speciality*” (MNT). They reported that friendships suffered as competition intensifies:

Our friendship is also reduced much when my friends are appointed to be my managers or that they are sent to study to get higher qualifications, while I do not get this although they are not smarter than me (PAYT).

The women academics who participated in the study believed that, due to intense competition, relationships at work were not as they should be and therefore, this impacted their working environment as well as life outside work. The working environment lacks support among colleagues and sharing of information. A typical comment is:

In relations at work, competition is very high, because lecturers must try to teach well and they also control information, they do not want to exchange information relating to teaching; they just want to work separately, they do not cooperate, and they only do for themselves, they keep their own information and do not share information with each other (VTDT).

The participants considered their relationships with peers as formal and civic, but competition between them was fierce. They described this as “*compete with each other silently*” (HNT) because the participants were not expected to speak out in order to keep harmony in their units and institutions, in accordance with the Confucius culture

Every one does their assigned work, this creates competition quietly, and it inhibits development (VTDT).

The reason for this as they indicated was secretive of the Vietnamese who often controlled information for themselves, as a woman stated:

The Vietnamese are conservative people, they hold information, they do not listen to others, and they just follow their belief (VTDT).

The interview different groups of women academics competed for different reasons. For instance, the competition among assistant lectures was to have job security, while young women lectures competed in trying to achieve higher qualifications and climb up to get into management positions through teaching and research. As for the old and senior lectures, they were more likely to compete in moving up to hold senior-level management positions. This is apparent in the three following quotes:

Competition in my group is high, especially for young faculty members as they strive for achieving certain degrees, and demonstrate their professional capabilities (TVH, lecturer)

**Fig. 3:**
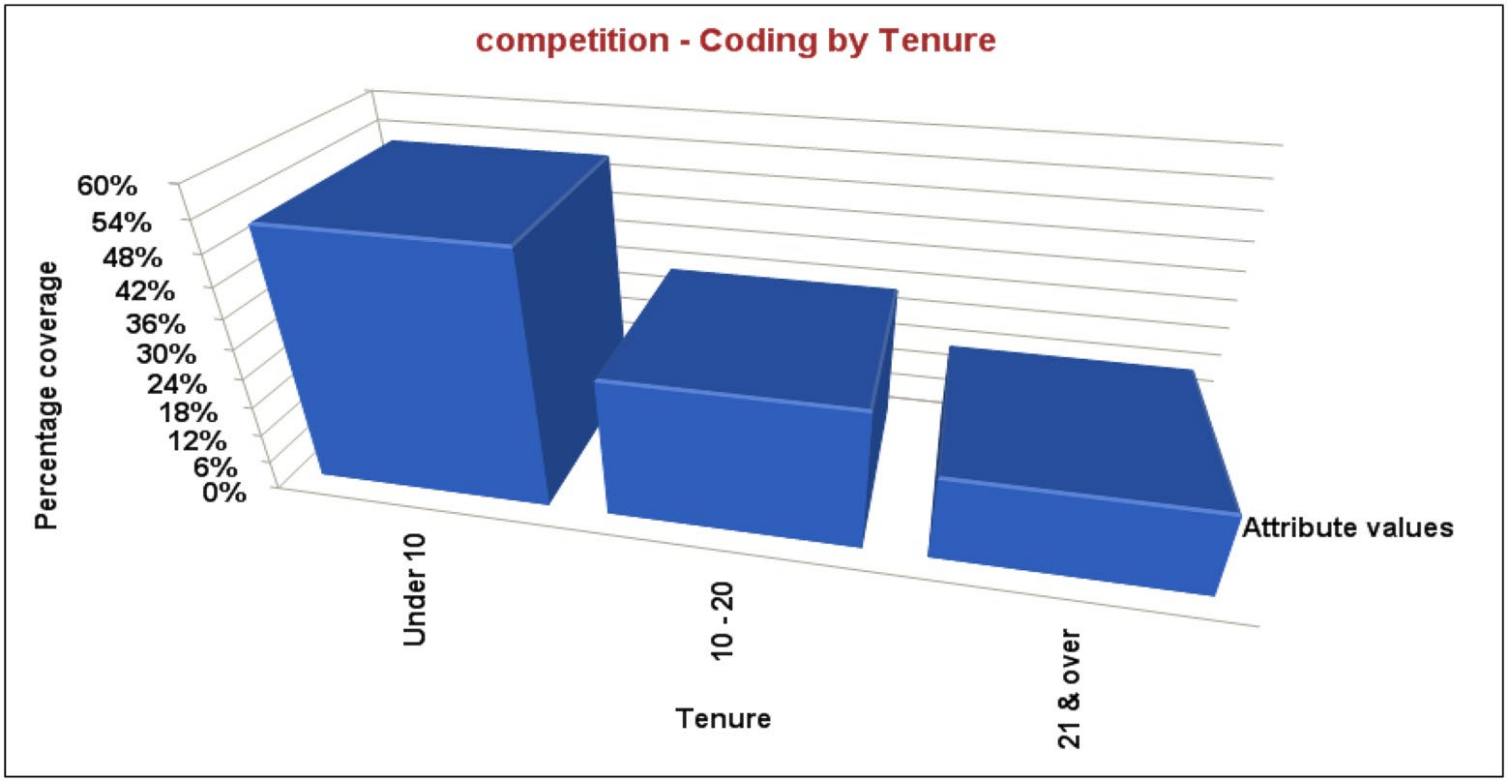
Indicates that occupational stress from competition is the highest among academics with less than 10 year experience and the lowest among women with over 21 year experience

The Fig. 3 indicates that occupational stress from competition is the highest among academics with less than 10 year experience and the lowest among women with over 21 year experience.

### Envy and jealousy

Envy, pettiness and jealousy are key factors in causing occupational stress on the academic women. This issue can be illustrated by their statements:

Of course, the competition among female lecturers is high and I think it is likely higher than male lecturers among themselves. Female lecturers are often jealous of the difference in profession and family (KNT).

The successes of their peers in similar age groups made some women stressful and affected their friendship at work.

Our friendship between the two is also much reduced. I also think differently about my managers who appoint my female friends or send them to study (PAYT).

However, from the point of those women who succeeded in gaining higher-level positions, they reported that they were also stressed because their colleagues were jealous of their successes.

I am stressed from my success, because envy makes many people dissatisfied, and because they are not successful like me, sometimes I’m depressed. Because I am successful in my work, but I am not always successful from public opinions, I think the more you are successful, the greater you are stressed (PCD).

Participants revealed that jealousy among peers was the main reason that caused stress on them and it was intense so much as that they were unhappy with their relations with their colleagues, for example: “*I am stressed from jealousy of my colleagues, this competition is unfair, and this stress is high”* (TCD).

### Some additional factors affect the stress caused by relationships

#### Educational qualifications

In addition to the factors causing OS in academic women in Vietnam as discussed above, underachievement (judged by level of attainment of managerial positions corresponding to qualifications) is also a source of OS. In the Vietnamese context, if a woman had a high degree, but she wasn’t appointed at a high position in management, people thought that she had no competence in her industry and expertise. Therefore this problem causes occupational stress on women with higher degrees, but they don’t have a ‘deserving’ position relative to their qualification, as a participant complained:

I am sad because I don’t have a role of management at all, because my friends ask me what role in management I play in order to evaluate my abilities and that is stress because they suppose that people with advanced degrees, and high knowledge must hold top management positions, and get promotion in their career. I do not speak out, but I find me bad and embarrassed when my friends or relatives ask me what position of management I hold” (TCD).

These relationships seemed to be more critical in cases where managers have lower degrees and competence in expertise than their subordinates. A typical expression was:

“My success has also created stress for other people, colleagues, especially managers who have lower degrees than me, but they manage me; the jealousy makes the relationships greatly stressful…. Their expertise is not as good as my one” (PCD).

Furthermore, this could also cause insecurity in managers with lower qualifications than the subordinates and as a result, relationships are put at risk.

“I’m under the authority of a person whom I don’t admire and who has a low degree, bad way of communication, no talent, and no morality, so I feel that I am offended and humiliated. …..I am trying to maintain, but they try to break necessary relationships because they are afraid of losing their safety on managerial positions…. They feel uncertain of their degrees and they don’t have relations with other people as well as I do” (TCD).

**Fig. 4:**
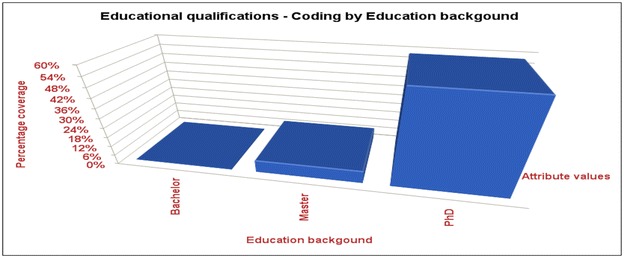
More academics with higher qualifications feel stressed than the rest with lower qualifications

The results show that more academics with higher qualifications feel stressed than the rest with lower qualifications illustrated in the Fig. 4.

#### Whether the academic was previously a student of her colleagues

Relationships between teachers and students in the Vietnamese hierarchical culture is a critical feature, by which students have to respect their teachers throughout the students’ lives, regardless of what they might become later in life. For instance, if a superior happens to be the subordinate’s student previously, this causes enormous discomfort in both their relationships. It means there was a contradiction between relations based on an ethical standard in the Confucian culture (lecturer-student) and relations based on power by law (manager-subordinate) and this caused considerable stress on her because she met many difficulties in her work, especially in her role as a manager as she expressed: “*Stress from colleagues’ relationships is high; especially I used to be their student***”** (MNT).

Those who used senior lecturers’ students believed that relationships with older colleagues at work was very difficult, affected their management and making-decisions and they were a significant source of stress because of hierarchy in Vietnamese culture, MNT went on: “*I have many difficulties in management and making decisions”*. In these relationships, in reverse, those who were colleagues’ lecturers did not felt these relationships stressful as they were respected by their students although now they were colleagues at the workplaces:

I am stressed from relationships at work, but, I don’t feel stressful from relationships with my colleagues in the unit, because most of them are younger than me and were my students (KCD).

It is acknowledged that relationships at work in Vietnamese culture play an essential role in experiencing occupational stress by Vietnamese academic women who consider that to maintain and cultivate harmonious relationships with colleagues is necessary to have a good standing in their work. However, this has also been a cause of occupational stress for many of them because of colleagues’ envy, pettiness and jealousy among women academics.

#### Confrontation

It is important to explore how participants expressed coping with occupational stress by confrontation with interpersonal conflicts in their relationships at work and Vietnamese culture may have impacts on the forms of expressions. Confrontation was understood by a majority of the participants in terms of reactions with conflicts in relationships at work when they got in trouble; and conflicts referred by them were ones mainly with their superiors (seniors and managers) who had power in Vietnamese culture, as respondent alleged that she was ready to confront with her superiors when she felt stress from problems in her workplace.

I confront directly with the seniors and my managers when I encounter problems. I am not afraid of dealing with them, for example, I refused to teach a course that was not related to my expertise (for testing), although I knew that the manager felt surprised, because subordinates often were ready to receive tasks assigned by managers (PAYT).

The research data have pointed to possible ways in which culture can affect the participants’ choice of direct and indirect confrontation or acceptance and also shed light on how the participants in Vietnamese culture experience occupational stress from this problem.

#### Direct confrontation

It is interesting to note in the study that many participants were more likely to confront directly with problems relating to relationships at work, basically relationships with their seniors and managers, than to confront indirectly or accept conflicts. Although relationships are considered one of the most important cultural traits in Vietnamese culture and it is necessary for them to cultivate the relationships with all people at work in order to have good opportunities and standing in the workplace, it does not mean that direct confrontation would destroy these relationships with the following reasons.

Firstly, older participants and participants with more experience, higher qualifications or with management responsibilities were ready to confront directly with interpersonal conflicts because they had power embedded in two concepts: seniority and authority, by which older persons and persons with higher status were considered to be more powerful and knowledgeable. The hierarchical values and orders of relationships and social stratification made them not be afraid of having direct confrontation and the ways of the confrontation may be a hidden motive to preserve harmony and save “face” because they were older and more senior.

If I have problems or conflicts with managers, I am ready to debate with them, before I dared not do it, but today I and they have worked together for a long time; now young female faculty are afraid of and dare not to confront with me (TVH).

**Fig. 5:**
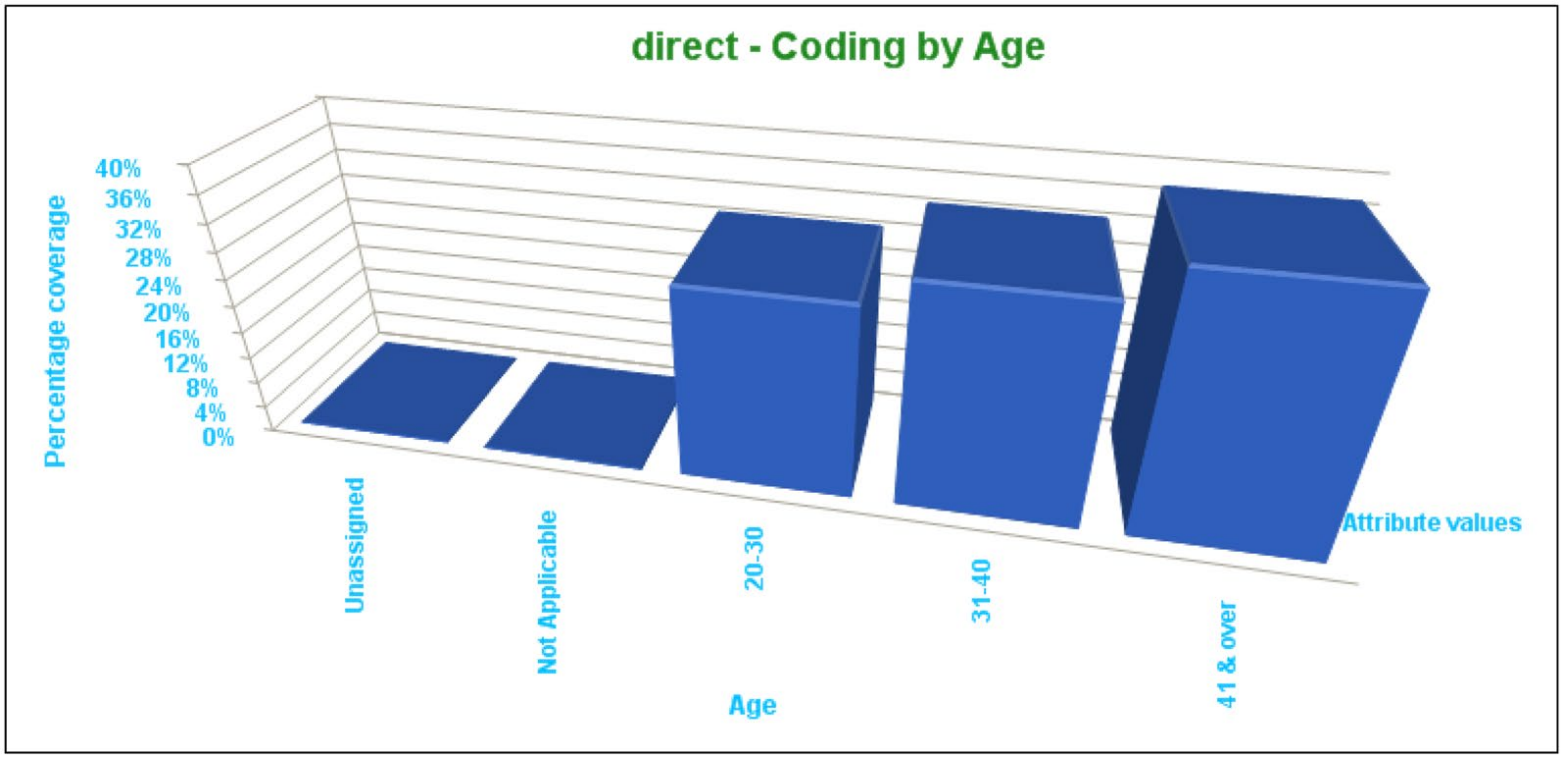
Women who are older use more direct confrontation as a coping strategy

The Fig. 5 asserts the above result, that is, women who are older use more direct confrontation as a coping strategy.

If participants, subordinates, were older or the same age with their managers who had more authority and power, they were not scared to confront directly because they had power of hierarchy and orders: older–younger; however, they were afraid of direct confrontation when they were younger

Before I was timid because I was young and I was not strong, but now I’m ready to confront with them. I’m not afraid of them and I myself escape (VACD).

Personality had an important factor having influence on direct confrontation. Those who had strong characteristics were ready to confront directly with their managers; and in reverse, managers who were sociable made it easier for their subordinates to confront with them directly.

Confrontation depends on the way of their management if they are authoritarian, patriarchal, I am afraid of confrontation, because this is not conducive, or through the third persons. If managers are sociable, I feel easy to debate and argue with them (PCD).

Direct confrontation was difficult and risky as some participants realized that this confrontation could hinder their career development and their managers did not like them. The value of conformity and obedience may help to maintain harmonious relationships with the superiors, without it the relationships became difficult:

I have conflicts with them, I dare not confront; because if I confront with them, I do not have any opportunities to develop, and consequences are unavoidable, because it is our culture (HNT).

Some participants engaged in it to help to resolve occupational stress, but they did this very carefully by always respecting their superiors and hierarchical structure, expressing being inferior to their superiors, inhibiting their negative emotions and forbearing consequences:

I am frank, this may be good but it also gives me a lot of heavy loss, fatigue and even great consequences. I don’t need the help from others; I usually confront with them directly by my sincerity in order to convince them. If I can’t persuade them, I use arguments against them, I accept the worst consequences. Resolving stress between me and the leaders are sincere, humble, so that they realize that I am only a subordinate and I am not equal with them. This let them see their important position. I don’t do anything to offend them, but if I can’t persuade them, I must use strong arguments and I accept any consequences because I can’t decide anymore (PCD).

These participants indicated that direct confrontation was the best way to resolve conflicts in relationships with the superiors at work; and the ways of this confrontation were diverse:

With the seniors, sometimes I have to accept, because when I explain, then they do not sympathize and share. And if it is necessary, I confront with them, I respond to resolving problems (TTBD).

Apparently, direct confrontation was chosen by many participants to resolve conflicts with others, especially, with their superiors. The research data indicate the cultural factors which had influences on the confrontation were ages, hierarchical orders, power, and partly their and their superiors’ personality. However, used direct confrontation was not to threaten relationships between them; although this was not conducive to them, it was expected to please and understand each another in order to resolve some problem in their relationships at work rather than to damage harmony:

Relationships at work are clear; I often confront directly to clarify problems. If I lack experiences, I learn; if it is necessary, I clarify to understand each others. I am humble and yielding to my managers. But if I have problems which affect my work, I confront to clarify (HCD).

#### Indirect confrontation

As mentioned above, many participants did confront directly, perhaps because they were older or had seniority; but others did not, due to risks, so they used another strategy of copping with occupational stress: indirect confrontation. The research participants referred to indirect confrontation in terms of the third parties:

I am afraid of the seniors. I don’t directly confront with my managers but I ask the third person to help me (GCD).

The importance and goal of maintaining harmonious relationships in their work life led to indirect confrontation in order to achieve ultimate goals of work, as losing the relationships made them difficult in their job as some participants said:

When I confront with the managers, I ask a third person to help me, because I dare not confront directly and it affects the relationships with them, when the relationships reduce, they treat me much worse, they criticize my work mistakes and I have trouble at work. If they do not like me, they consider me a bad person (VACD).

**Fig. 6:**
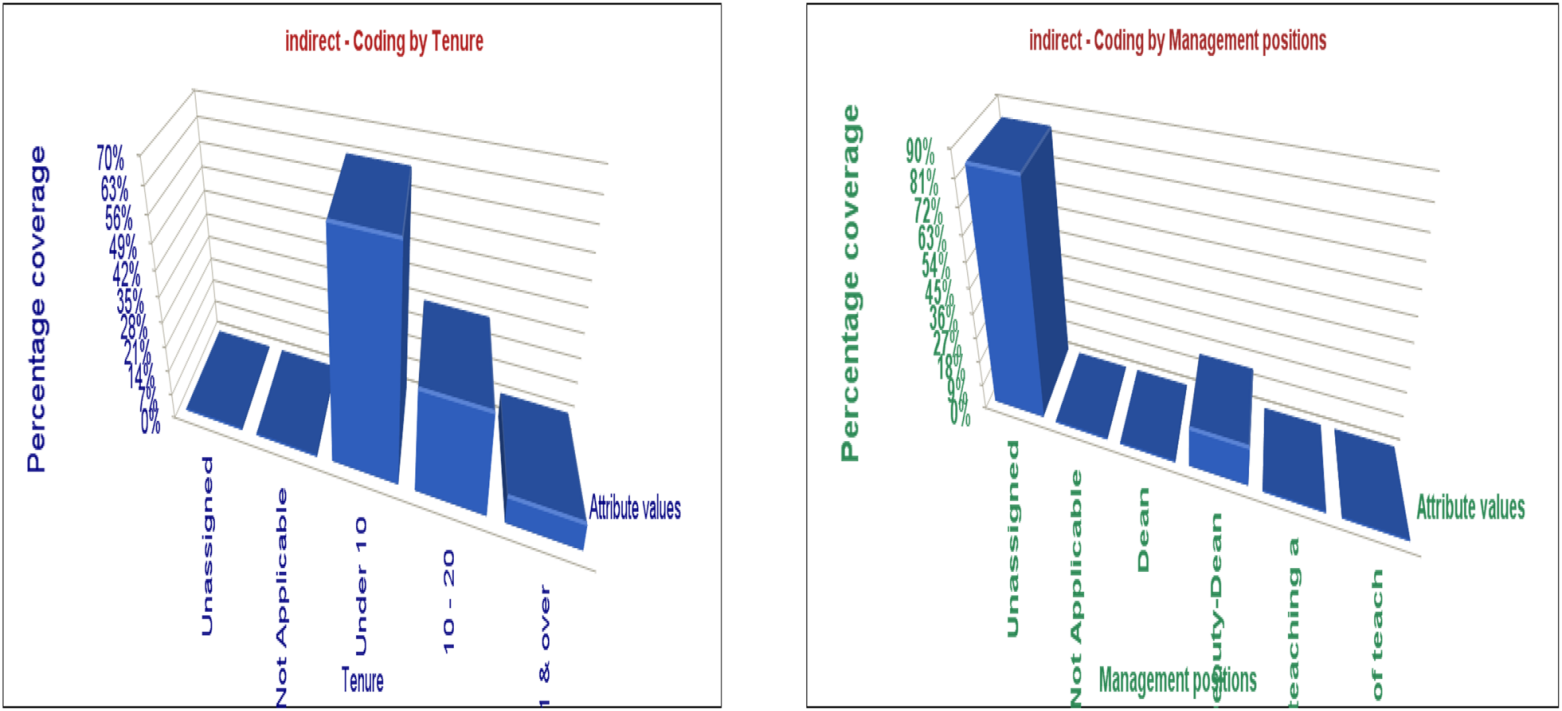
Academic women with lower experience and no managerial positions are likely to use indirect confrontation more frequently than others as illustrated in the

According to them, indirect confrontation was more conducive and effective than others; and involving third parties would lead the participants to avoid the risks in resolving stress from conflicts with their superiors. Academic women with lower experience and no managerial positions are likely to use indirect confrontation more frequently than others as illustrated in the Fig. 6.

As mentioned by some participants below, ways of indirect confrontation were consistent with involving third parties in order to maintain the relationships in which parties were viewed as embedded. Moreover, power and hierarchy in Vietnamese culture made them scared, so the best third person was a powerful person with management responsibilities and the best ways of confrontation were:

I have no direct confrontation with the managers, because they have power to decide, I must respect their opinions. If I am stressed with my managers, I ask others for help (TrVH).

**Fig. 7:**
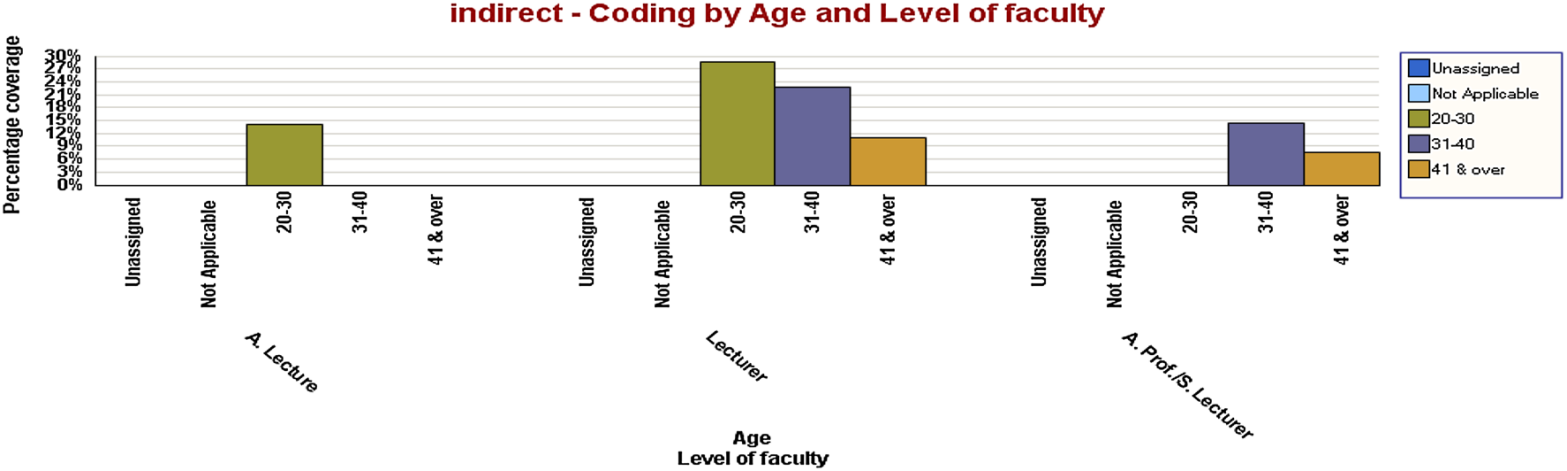
Indicates relationships of indirect confrontation between ages and level of faculty, for example, younger lecturers were likely to use indirect confrontation more frequently than the older

The Fig. 7 indicates relationships of indirect confrontation between ages and level of faculty, for example, younger lecturers were likely to use indirect confrontation more frequently than the older.

It can be seen that some of the study participants chose more direct confrontation in resolving conflicts in relationships with their superiors than others, but the characteristics of Confucianism were maintained in the ways they confronted directly. The higher status and the lower status parties did not harm their relationships because they did not want to show disrespect and offense to others. It is realized that most of participants who were ready to confront directly were older and more powerful because they also have power of seniority and authority in hierarchical culture; while other groups of women were likely to prefer expressing acceptance and confrontation in indirect ways with the help of the third parties, especially party holding positions of management in order to maintain the relationships.

#### Consequences

Most of the participants reported that occupational stress from relationships at work made consequences on them negative; personally, majority of women subordinates reported that relationships at work hinder their individual development as a consequence of occupational stress because they “*have to do what their managers and seniors instruct*” (TrYT) and this led to obstruct their creativity in the job.

Although the college tries to establish and create good relationships, models of hierarchical relationships restrict creativity (KVH)

Their success or failure was decided by these relationships, which were more important than expertise, as a woman expressed:

Expertise is secondary, compared to relationships, if they maintain good relations and communication, they have more opportunities (HTBD).

The participants provided interesting explanation of the role of Vietnamese culture on their relationships. They identified the “crab” culture and “gift” culture and indicated both have impacts on the stress they experienced in their relationships at work with their colleagues and superiors. Outcome of occupational stress from these relationships resulted if, due to this cultural belief, subordinates were always expected to be inferior to their superiors and were constrained in their abilities to advance.

Vietnamese culture is a “crab” culture…., compared with western cultures, it can be seen that role of leadership in Western countries is only a little higher than role of others who are leaders’ peers, but much higher than that in Asian countries; in Vietnam, leaders are giants, subordinates are tiny, the role of leaders is too large and they lead by their wills, and they are willing to impede factors under their authority. They do not want their subordinates better than them and these problems make management stagnant (TCD).

Moreover, “gift” culture also revealed that “*leaders know that their subordinates are interested in them and afraid of them*” (HNT). Another consequence of occupational stress from the relationships at work is the fact that “*the older do not want the younger to be their managers*” (TVH) due to hierarchy in Vietnamese culture, in which the younger have to respect the older because:

Given the fact that in Vietnamese culture, the order in relationships is very important and if somebody destroys these relationships, of course, they have difficulties and bad consequences in their career (KNT).

In reverse, younger managers and managers who were the previous students of the institutions met “*difficulties in making decisions”* (HTBD); moreover, women peers also were hard to attain their work goals due to their peers’ envy, jealousy and competition, as a woman said:

If I am better than them, they are jealous of my success; if I’m worse than them, they will show contempt for me; if I am as good as them, they will envy me from the smallest things (VACD).

Academic women subordinates using direct confrontation could experience both loss, such as career development, relationships and job hindrance; in reverse, women that had acceptance and indirect confrontation with conflicts in relationships at work felt their stress more severe, if issues couldn’t be resolved, because confrontation was impossible, occupational stress stayed high:

I’m afraid of confronting with them. I always show the comfort and warmth, but I am not satisfied (TAYT).

Apparently, occupational stress from relationships at work influences participants’ career, especially career of the younger and subordinates due cultural factors: hierarchy, power distance and respect. Relationships are more important than speciality and expertise, without them participants have job hindrance in their career.

## Discussion

This study contributes to the occupational stress literature by exploring how cultural factors influence occupational stress originating relationships at work. The research results support the findings of research by Etzion and Pines [16], Glazer and Beehr [19], Liu et al. [28], Idris et al. [22] and Bahrami [3] that culture is a key factor in identifying occupational stress and the coping mechanisms used by individuals when they are confronted with stressful events; however, key studies by these researchers distinguish only two cultural factors: individualism and collectivism. Although the construct of individualism and collectivism has played a central role in cross-cultural research on occupational stress, there are many cultural differences within the set of countries included in these two broad groups that need also to be explored. Furthermore, the focus on individualism and collectivism tends to ignore a range of other cultural variables that also affect occupational stress, such as power distance based on hierarchy at work and age and gender roles [15, 44].

The evidence compiled in this paper shows that managers and older individuals in Vietnamese universities are accorded power and respect and are not expected or encouraged to share their power and experiences with their subordinates or younger people more generally. As a consequence, academic women in subordinate positions typically expect to be told what to do; whilst managers are expected to lead and resist self-management.

The effect of power distance on the occupational stress experienced by younger women academics is particularly strong as there is a gap between both superiors and subordinates and between older and younger individuals. In some instances, the cultural respect that is accorded to older individuals obstructs the career opportunities of younger women. Reflecting this, this study finds that many younger women academics, compared with the older, experience occupational stress in Vietnamese universities.

The importance of the older-younger hierarchy in determining the occupational stress experienced by young women in Vietnamese universities is evidenced by findings from this project that show that young academic women managers experience occupational stress even when they have power in their organisational role. In Vietnam, older individuals have virtuous power and respect for age is a cardinal virtue [36].

The older–younger cultural hierarchy is also relevant to the occupational stress experienced by older women academics in Vietnam. This is apparent in situations where they are managed by younger individuals or, indeed, by their peers. It is not generally accepted that individuals of the same age and status are entitled to be appointed to superior positions—and this can result in envy and competition. In accordance with Napier’s [31] more general observations, the age-based hierarchy makes it difficult for individuals of the same age and status to become managers of their peers. Relationships among colleagues are also affected by what is referred to as culture “crab”, in which nobody wants to be inferior to others and others do not want anybody to be better than them (see also [32]).

This study finds that superior-subordinate hierarchy can be a particular source of occupational stress for academic women who have higher education qualifications than their managers, but perceive that they have to obey and accept any tasks assigned by him or her. Some women in these situations do not dare to reveal their capacities or their dissatisfaction with assigned tasks because they perceive that they must maintain their relationship with their manager in order to attain career achievement. As Napier [31] also describes, the Vietnamese must accept seniority for their career development.

The effort involved in maintaining relationships with superiors is an associated source of stress for Vietnamese academic women. The women often perceive that they must make themselves appear inferior to their superiors to maintain the power-distance required by the cultural hierarchy, regardless of their qualifications and capacities, and this hinders the development of all aspects of their work. Stress also results from the inequity that is perceived when individuals who have good relationships with their superiors, but do not have capacities and high qualifications, have the greatest career opportunities. This finding is in accordance with those Zhang [45], Berscheid and Reis [4] and Liu and Spector [26].

The fear of losing ‘face’ (the individual’s public image) and the importance attached to keeping harmony in the workplace were important other sources of occupational stress identified in this study and related to the cultural environment of Vietnamese universities. Most of the research participants in this study reported that they were afraid of losing their own face in front of their colleagues and especially in front of their students if direct conflicts are addressed, anger is demonstrated and/or criticisms are revealed. In accordance with Hofstede and Hofstede’s [21] more general observations of Vietnamese culture, losing face was perceived to be a potential source of serious personal damage and something one should try to avoid at any price (also see [13]).

## Conclusion

Cultural relationships are considered as the stress experienced by Vietnamese academic women, as they impact on work relationships. Occupational stress at work declines for Vietnamese academic women as they advance in age, experience, rank and qualification. This is because the key relationships referred to above: between the older and the younger, between the subordinate and manager, and between the student and lecturer start to move in their favour as the women age. Relationships in Vietnamese culture are important; and this is a key source of occupational stress at work for academic women.

## Limitations and suggestions for future research

The results of this paper need to be viewed in the light of the research’s limitations. One of these is the absence of quantitative data to augment the qualitative results of this study. The quantitative measures to supplement the use of interview data in future studies could provide a more in-depth picture of the phenomenon of occupational stress at work among Vietnamese academic women. Specifically, a large scale quantitative study could provide measures of relationships between key demographic and other variables and the level of occupational stress. Such data would complement the findings of the current study by measuring the degree of occupational stress which women experience and the sources of variation in these levels.

## Abbreviations

OC: occupational stress
VN: VietNam
MoET: Ministry of Education & Training

## References

[1] Altbach GP, Reisberg L, Rumbley LE. Trends in global higher education: tracking an academic revolution. In: UNESCO 2009 world conference on higher education. France: The United Nations Educational, Scientific and Cultural Organization. 2009.

[2] Anderson ML (1997). Thinking about women: sociological perspectives on sex and gender.

[3] Bahrami, S. (2010). Influences of Culture and Social class on perception of job Stress in emerging economies. *International Review of Business Research Papers*, 6(2): 52-67. URL: http://www.bizresearchpapers.com/4.Shahin.pdf.

[4] Berscheid E, Reis HT, Gilbert DT, Fiske ST, Lindzey G (1998). Attraction and close relationships. The handbook of social psychology.

[5] Bringer JD, Johnston LH (2004). Maximizing transparency in a doctoral thesis1: the complexities of writing about the use of QSR*NVIVO within a grounded theory study. Qual Res.

[6] Chalmers A (1998). Workload and stress in New Zealand universities: a follow-up to the 1994 study.

[7] Christie MD, Shultz KS (1998). Gender differences on coping with job stress and organizational outcomes. Work & Stress.

[8] Corbin J, Strauss A (2008). Basics of qualitative research: Techniques and procedures for developing grounded theory.

[9] Creswell WJ. Educational research: planning, conducting, and evaluating quantitative and qualitative research, Pearson. 2008.

[10] Deem R, Brehony KJ (2005). Management as ideology: the case of ‘new managerialism’ in higher education. Oxford Rev Educ.

[11] Denzin NK, Lincoln YS (2000). *Handbook of Qualitative Research*.

[12] Dodge J, Ospina SM (2005). Integrating rigor and relevance in public administration scholarship: the contribution of narrative inquiry. Public Adm Rev.

[13] Esmond et al, 1996 . Doing Business in Vietnam .*Business Horizons* 29(3):

[14] Elliott M (2008). Gender differences in the causes of work and family strain among academic faculty. J Hum Behav Soc Environ.

[15] Essau CA, Trommsdorff G (1996). Coping with university-related problems: a cross-cultural comparison. J Cross Cult Psychol.

[16] Etzion D, Pines A (1986). Sex and culture in burnout and coping among human service professionals: a social psychological perspective. J Cross Cult Psychol.

[17] Fisher S (1994). Stress in academic life: the mental assembly line.

[18] Frese M, Zapf D, Dunnette MD, Hough LM (1994). Action as the core of work psychology: a German approach. Handbook of industrial and organizational psychology.

[19] Glazer S, Beehr TA (2005). Consistency of Implications of Three Role Stressors across Four Countries. J Org Behav.

[20] Hart JL, Cress CM (2008). Are women faculty just worrywarts? Accounting for gender differences in self-reported stress. J Hum Behav Soc Environ.

[21] Hofstede G, Hofstede JG (2005). Cultures and organizations: software of the mind.

[22] Idris AM, Dollard MF, Winefield AH (2010). Lay theory explanations of occupational stress: the Malaysian context. Cross Cult Manag Int J.

[23] Iwasaki Y, MacKay KJ (2004). Gender-based analyses of stress among professional managers: an exploratory qualitative study. Int J Stress Manag.

[24] Jick TD, Mitz LF (1986). Sex differences in work stress. J Libr Admin..

[25] Kikooma FJ (2010). Using qualitative data analysis software in a social constructivist study in entrepreneurship. Qual Res J.

[26] Liu C, Spector PE, Barling J, Kolloway K, Frone M (2005). International and cross cultural issues. Handbook of work stress.

[27] Liu CS, Cooper PE, Shi L (2007). Cross-national job stress: a quantitative and qualitative study. J Organ Behav.

[28] Liu C, Nauta MM, Spector EP, Li C (2008). Direct and indirect conflicts at work in China and the US: a cross-cultural comparison. Work Stress.

[29] Michailidis PM (2008). Gender-related work stressors in tertiary education. J Hum Behav Soc Environ.

[30] MoET (2009, 2012). http://www.moet.gov.vn

[31] Napier KN (2005). Knowledge transfer in Vietnam: starts, stops and loops. J Manag Psychol.

[32] Napier NK, Thomas DC (2004). Managing relationships in transition economics.

[33] Narayanan L, Menon S (1999). A cross-cultural comparison of job stressors and reactions among employees holding comparable jobs in two countries. Int J Stress Manag.

[34] O’Laughlin EM, Bischoff LG (2005). Balancing parenthood and academia: work/family stress as influenced by gender and tenure status. J Fam Issues.

[35] Sanchez JI, Spector PE, Cooper CL, Wong PTP, Wong LLJ (2006). Frequently ignored methodological issues in cross-cultural stress research. Handbook of multicultural perspectives on stress and coping.

[36] Smith ED, Pham C (1996). Doing Business in Vietnam: A Cultural Guide. Business Horizons.

[37] Siccama JC, Penna S (2008). Enhancing validity of a qualitative dissertation research study by using Nvivo. Qual Research J.

[38] Strauss A, Corbin J (1990). Basics of qualitative research: grounded theory procedures and techniques.

[39] Strauss A, Corbin J (1998). Basics of qualitative research: Techniques and procedures for developing grounded theory.

[40] Thanacoody PR, Bartram T, Barker M, Jacobs K (2006). Career progression among female academics. Women Manag Rev.

[41] Tytherleigh MY, Jacobs PA, Webb C, Ricketts C, Cooper C (2007). Gender, health and stress in English University staff; exposure or vulnerability?. Appl Psychol.

[42] Vagg PR, Spielberger CD, Wasala CF (2002). Effects of organizational level and gender on stress in the workplace. Int J Stress Manag.

[43] Winefield AH, Boyd C, Saebel J, Pignata S (2008). Job stress in University staff.

[44] Wong PTP, Wong LCJ (2006). *A Handbook of Multicultural Prospectives on Stress & Coping*.

[45] Zhang L (2010). A study on the measurement of job-related stress among women academics in research universities of China. Front Educ China.

